# Content validity of a newly developed observer-reported measure for pediatric asthma in children aged 2–5 years

**DOI:** 10.1186/s41687-022-00461-y

**Published:** 2022-05-28

**Authors:** Yi Zhang, Jennifer L. Clegg, Shannon Keith, Shehan McFadden, Tara Symonds, Rajesh Kumar, Asif H. Khan, Siddhesh Kamat, Jingdong Chao

**Affiliations:** 1 Regeneron Pharmaceuticals, Tarrytown, NY USA; 2 Clinical Outcomes Assessment, Clinical Outcomes Solutions, 53 W Jackson Blvd, Suite 1150, Chicago, IL 60604 USA; 3 Clinical Outcomes Solutions, Folkestone, UK; 4 Lurie Children’s Hospital, Chicago, IL USA; 5 Sanofi, Chilly-Mazarin, France

**Keywords:** Pediatric Asthma Questionnaire, PAQ, Content validity, Health-related quality of life, HRQoL, Patient-reported outcome, PRO, Observer-reported outcome, ObsRO, Pediatric, Asthma

## Abstract

**Background:**

An observer-reported outcome (ObsRO) measure assessing both symptom control and health-related quality of life (HRQoL) in children with asthma younger than 6 years is lacking. The objective of this study was to evaluate the content validity of the Pediatric Asthma Questionnaire (PAQ), a newly developed 6-item ObsRO measure for caregivers of children aged 2–5 years diagnosed with asthma.

**Results:**

In-depth, qualitative interviews were conducted with 15 parents or caregivers. The first part of the interview was an open-ended discussion whereby participants were asked to describe their observations of their child’s asthma symptoms and HRQoL impacts followed by a cognitive debriefing of a draft version of the PAQ. The most frequently reported symptoms were coughing (n = 15, 100%), wheezing (n = 14, 93%), and trouble breathing (n = 10, 67%). Overall, participants found the PAQ easy to complete and relevant to their child’s experience with asthma, with most reporting the instructions, response scales, and recall period for the items to be appropriate. The majority of participants (93%) believed they could accurately report on the items included in the PAQ based on their observations of their child’s asthma symptoms and impacts, or reliably get the information from the child’s teacher, school, or caregiver when their child was not in their presence. One item was modified based on feedback about the phrase “oral steroids” to clarify modes of administration. A few other minor changes were incorporated into the PAQ following suggestions from participants, including replacing the phrase “how often” with “how many days” in one of the items to improve clarity and overall consistency with the response options.

**Conclusion:**

Qualitative data support the content validity of the PAQ as a fit-for-purpose and well-understood 6-item observer-reported outcome measure to evaluate both symptoms and asthma-specific HRQoL impacts experienced by pediatric asthma patients aged 2–5 years for use in clinical and real-world studies.

## Introduction

Childhood asthma is a chronic condition characterized by symptoms including wheezing, shortness of breath, tightness in the chest, and coughing. According to the most recent data from the Centers for Disease Control, in 2019 asthma prevalence in children (age < 18 years) was approximately 5.1 million (7.0%) in the United States (US) [1] and is the most common chronic disease among this age group. Childhood asthma is a common cause of emergency room visits, hospital admissions, school absences, and parental work absenteeism [2]. It is a leading cause of school absenteeism and accounts for more than 13.8 million missed school days [3]. Asthma disproportionately affects socioeconomically disadvantaged and minority children, with higher prevalence rates and worse outcomes [4]. Recent data shows the incidence of pediatric asthma among children aged 0–4 years was 23.4/1000, more than 5 times greater than that among youth aged 12–17 years (4.4/1000) [5].

As more treatments and interventions are being developed, clinical trials often incorporate patients’ self-report of symptoms, known as a patient-reported outcome (PRO) measure or instrument, to evaluate symptom and health-related quality of life (HRQoL) assessments from the patient perspective. If the patient is too young or otherwise cannot report for themselves, an observer-reported outcome (ObsRO) is used. ObsROs rely on a parent or caregiver to objectively report on observable signs, symptoms, and behaviors related to the patient’s condition.

Currently, there is a wide range of pediatric asthma PRO instruments that measure symptoms and HRQoL impacts for children aged 6–18 years [6–11]. However, for children with asthma aged 2–5 years, existing pediatric asthma questionnaires have several shortcomings that limit their use in both clinical trial and practice settings. These include lengthy recall periods and/or a reliance on parent or caregiver proxy reporting rather than reports of directly observable signs and symptoms the child may be experiencing [12–17]. Considering the young age of patients and variability in capacity of children under the age of 8 years to report on their health accurately [18], an ObsRO was developed to meet the need to evaluate asthma symptoms and impacts efficiently in clinical trial and clinical practice settings. The Pediatric Asthma Questionnaire (PAQ) is a 6-item ObsRO measure for parents or caregivers of children aged 2–5 years with clinician-diagnosed asthma, or those with recurrent viral-associated wheezing with risk factors for persistent asthma, to assess both symptom control and asthma-specific HRQoL impact. (As the diagnosis of asthma in children under age 5 is both difficult and controversial [19, 20], for the purposes of this study, we have incorporated a more inclusive definition of asthma to include those with viral-associated wheezing with risk factors for persistent asthma). The PAQ features two items that measure asthma exacerbations which use a recall period of “the past month” while the remaining asthma symptoms (coughing a lot, difficulty breathing, or noisy breathing from the chest) and asthma-specific HRQoL items use “the past week.” In this instance, HRQoL can be defined as the *functional* or *wellbeing*-related impacts as a result of asthma symptoms. It is expected that the PAQ will take no more than 5 minutes to complete.

Development of the initial draft of the PAQ involved an iterative process including an literature and instrument review, clinician interviews, and expert review which is supported by the 2009 Food and Drug Administration (FDA) Guidance for Industry on PRO measures [21]. The literature and instrument reviews for children with asthma were conducted in previous work to generate qualitative content and review existing childhood asthma measures. Common symptoms and HRQoL impacts in pediatric asthma were summarized from the literature review. Three instruments focusing on symptom control and/or impacts in young children with asthma were reviewed: the PROMIS Pediatric Short Form v2.0—Asthma Impact 8a [22]; the Childhood Asthma Control Test (C-ACT) [17]; and the Test for Respiratory and Asthma Control in Kids (TRACK) [16]. Additionally, 2 in-depth, 90-min, one-on-one interviews were conducted with pediatric asthma clinicians to gain insights about the condition for this young age group and get feedback on relevant item concepts. Following the item draft development, the measure was reviewed by experts to refine the items and ensure it covered the most important and relevant symptom and impact concepts of pediatric asthma. For a newly drafted PRO or ObsRO measure, assessment of content validity with individuals who are to complete the measure is essential [21, 23, 24]. Content validity establishes that all concepts of interest from the patient perspective have been adequately captured in the measure, and that items and response options are worded in a way that is easily understood by the population who are to complete the measure. For this reason, a qualitative study was conducted with caregivers to evaluate the content and interpretation of the PAQ for use in children aged 2‒5 years with clinician-diagnosed asthma prior to moving to its psychometric evaluation.

## Methods

### Study design and participants

In-depth, semi-structured telephone interviews were conducted with caregivers of children aged 2–5 years with a clinician-reported asthma diagnosis. The interviews had 2 sections: (1) a brief open-ended discussion in which participants described observations of their child’s asthma-related symptom experience and impact on HRQoL; and (2) cognitive debriefing of the PAQ, which was designed to assess the relevance of the items and participants’ comprehension and interpretation of the items, response scale, recall period, and the ability of the observer to report accurate data for each item. The methods used in this study followed the FDA guidance regarding content validity of PRO and ObsRO measures to support labeling claims [21, 25] and was conducted in line with established research practices, including the guidelines provided by the International Society for Pharmacoeconomics and Outcomes (ISPOR) taskforce [23] and the Declaration of Helsinki and US 21 Code of Federal Regulations [26].

Participants met the following inclusion criteria: (1) being the parent, guardian, or primary caregiver of a child between the ages of 2–5 years with clinician-diagnosed asthma; (2) able to speak, read, and understand US-English; and (3) willing to give informed consent and be audio-recorded during the interview session. Participants were excluded if: (1) their child had a diagnosis of a chronic lung disease of prematurity, bronchopulmonary dysplasia, cystic fibrosis, had undergone thoracic surgery, or had a history of tuberculosis; (2) if their child had another serious chronic illness that the clinician believed would interfere with the caregiver’s ability to discern the symptoms of asthma; and (3) if they had cognitive, learning, visual, or speech disabilities.

Participants were identified using purposive sampling by a recruitment agency from 4 locations within the US: Chicago, Illinois; St. Louis, Missouri; St. Paul, Minnesota; and New Orleans, Louisiana. Once participants had been contacted and consented to being part of the study, the recruitment agency liaised with the site clinician to complete the Eligibility and Medical History Form and worked with participants to complete the Demographics Health Information Form. Once all forms had been completed, participants were scheduled for a one-on-one, 45-min telephone interview at their convenience and were mailed a copy of the PAQ questionnaire in a sealed enclosed envelope with instructions not to open until the scheduled interview time.

**Fig. 1:**
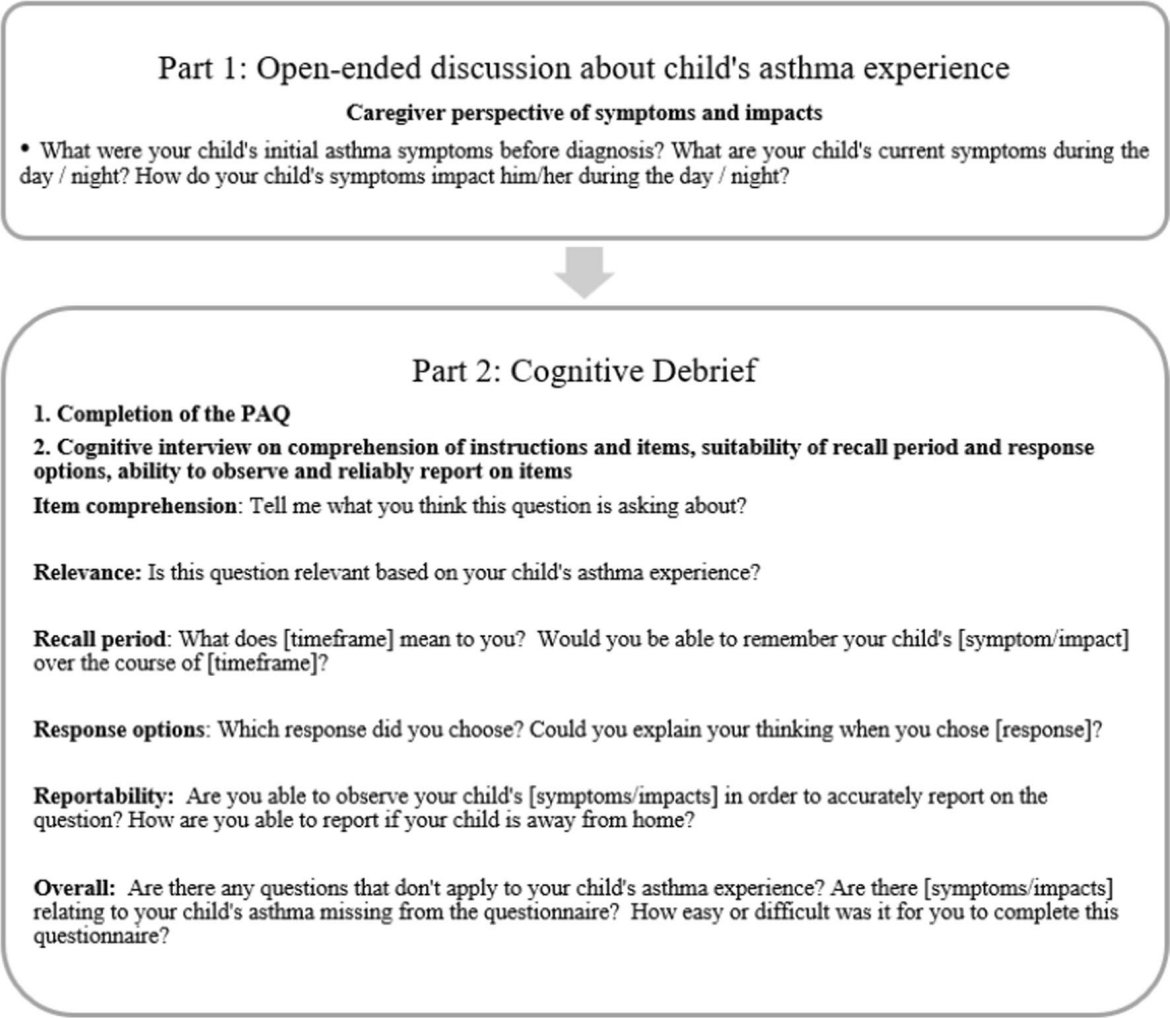
Summary of interview procedures

Interviews were conducted by 2 experienced qualitative researchers using a semi-structured, open-ended discussion and cognitive debrief interview guide. The interview procedures are summarized in Fig. 1. Interviews were audio-recorded, transcribed verbatim, and de-identified of personal information.

### Analytical approach

Participant demographic and medical history data were summarized to characterize the total sample and provide context to the qualitative data. Descriptive statistics (mean, median, standard deviation, and frequency) were calculated using SAS v9.4 software.

Two approaches were used for analyzing the 2 different types of interview data. The open-ended discussion data were analyzed using inductive thematic analysis [27]. Thematic analysis is a reflexive and iterative method for identifying, organizing, describing and reporting of themes found within a data set [27]. The objective of the open-ended discussion and analysis was to confirm that the symptom and impact concepts included in the PAQ were relevant for this population. Thus, a saturation analysis was performed on an individual concept level by dividing the sample into 3 sets of 5 participants (n = 5, n = 5, n = 5) in chronological order by the interview date. Saturation is achieved when no new relevant concepts emerge from the last set of participants. For the cognitive debrief data, a deductive content analysis approach was used, meaning, analysis focused on quotes that supported or contradicted the main research questions (ie, comprehension, concept relevance, comprehensiveness, and any rewording suggestions). Since the measure is an ObsRO, an additional research question about caregivers’ ability to observe and report accurately on each item of the PAQ (eg, when the child is not in the caregiver’s presence) was also assessed.

Qualitative coding of interview data was performed using NVivo PRO v12.0, a qualitative analysis software. To facilitate analysis of the interviews, a codebook was created based on the discussion guide. The codebook provides a basic coding framework from which researchers interpret the interview data based on the main research questions. Two coders met regularly to discuss and review codes and themes. Upon completion, a review was conducted to ensure codes had been applied accurately and consistently, and any discrepancies were adjudicated with consensus from the research team.

## Results

A total of 15 interviews were conducted with caregivers of children (7 males [47%], 8 females [53%]) with clinician-diagnosed asthma and were between the ages of 2–5 years. Complete sociodemographic and clinical characteristics are presented in Tables 1 and 2.

**Table 1:** Demographic characteristics of children and caregivers

Characteristic	Age 2–3 years n (%)	Age 4–5 years n (%)	Total n (%)
N	5 (33.3)	10 (66.7)	15 (100)
*Sex of child*			
Female	2 (13.3)	6 (40.0)	8 (53.3)
*Age*			
Mean (min–max)	2.60 (2.00–3.00)	4.60 (4.00–5.00)	
*Race of child*			
White	4 (26.7)	6 (40.0)	10 (66.7)
Black	0 (0)	4 (26.7)	4 (26.7)
Other	1 (6.7)	0 (0)	1 (6.7)
*Ethnicity of child*			
Hispanic/latino	1 (6.7)	2 (13.3)	3 (20.0)
*Sex of caregiver*			
Female	5 (100.0)	8 (80.0)	13 (86.7)
*Age of caregiver*			
Mean (min–max)	31.40 (26.00–38.00)	32.30 (23.00–41.00)	
*Caregiver education*			
High school diploma (or GED)	1 (6.7)	2 (13.3)	3 (20.0)
Some college	2 (13.3)	0 (0.0)	2 (13.3)
College or university degree	2 (13.3)	5 (33.3)	7 (46.7)
Graduate degree	0 (0.0)	2 (13.3)	2 (13.3)
Other	0 (0.0)	1 (6.7)	1 (6.7)

Demographics were caregiver-reported

**Table 2:** Clinical characteristics of children

Characteristic	Age 2–3 years n (%)	Age 4–5 years n (%)	Total n (%)
*Comorbidities*			
Allergic rhinitis	2 (13.3)	2 (13.3)	4 (26.6)
Chronic rhinosinusitis	0 (0.0)	0 (0.0)	0 (0.0)
Eosinophilic esophagitis	0 (0.0)	0 (0.0)	0 (0.0)
Nasal polyposis	0 (0.0)	1 (6.7)	1 (6.7)
Atopic dermatitis	0 (0.0)	2 (13.3)	2 (13.3)
*Exacerbations*			
Clinician-diagnosed asthma – 1 time in past month	1 (6.7)	1 (6.7)	2 (13.3)
Hospitalization – 1 time in past month	0 (0.0)	1 (6.7)	1 (6.7)
Severity			
*Caregiver rating of child’s asthma severity (last 7 days)*			
No symptoms	2 (13.3)	5 (33.3)	7 (46.6)
Mild	2 (13.3)	2 (13.3)	4 (26.6)
Moderate	0 (0.0)	2 (13.3)	2 (13.3)
Severe	1 (6.7)	1 (6.7)	2 (13.4)
Clinician rating*of child’s asthma severity (last 3 months)			
Very mild	1 (6.7)	0 (0.0)	1 (6.7)
Mild	1 (6.7)	3 (20.0)	4 (26.7)
Moderate	1 (6.7)	4 (26.7)	5 (33.4)
Severe	2 (13.3)	3 (20.0)	5 (33.3)
Current treatment**			
ICS	2 (13.3)	4 (26.7)	6 (40.0)
ICS + additional	2 (13.3)	4 (26.7)	6 (40.0)
SABA	4 (26.7)	7 (46.7)	11 (73.4)
Maintenance OCS	2 (13.3)	4 (26.7)	6 (40.0)

*ICS* inhaled corticosteroids; *OCS* oral corticosteroids; *SABA* short-acting beta agonist

*Made by the child’s asthma clinician

**Treatments were not mutually exclusive

### Symptom and impact results

The most commonly reported pediatric asthma symptoms by caregivers were cough (n = 15, 100%), wheeze (n = 14, 93%) and trouble breathing (n = 10, 67%). Other symptoms caregivers reported as asthma (or asthma-related) included sneezing, tightness in chest and runny nose, each mentioned by 2 caregivers. Notably, some symptoms reported by caregivers were not typical of asthma. All other symptoms were mentioned by only 1 individual (e.g., congestion, lethargy, watery eyes). The most commonly mentioned symptoms by caregivers are described in greater detail below.

All caregivers reported coughing as a symptom of their child’s asthma, which can occur both during the day (n = 8) and at night (n = 9). Caregivers’ descriptions of day-time coughing ranged from occasional coughing to intense coughing fits, as described by this caregiver of a 5 year-old girl: “*Sometimes she, she coughs so much that she vomits… if she's vomiting too much, then I call her pediatrician”.* Other caregivers described coughing fits brought on by “*running around*”, “*playing*” or catching a cold or respiratory virus. Caregivers also explained that coughing at night would significantly disrupt their child’s sleep, as explained by this caregiver of a 3 year-old boy: “…*he'd cough throughout the whole night, there'd be no wheezing but there'd be coughing, so he just wouldn't sleep well”*. Coughing was considered the “most bothersome” of their child’s asthma symptoms by (n = 9) 60% of caregivers.

Wheezing was another important symptom, which caregivers reported 93% of the time (n = 14). Many caregivers described wheeze in terms of being able to hear their child breathing (n = 9). For example, one called it “*noisy breathing”*, and another noted it was “…*like, um, noise inside his chest*”. One caregiver of 5 year-old boy stated, “*I could hear the wheezing, um, or sometimes it just sounds really, um, junky… like a lot of congestion”*. Some caregivers noted the symptom was more apparent when their child was more active, for example, “*Certain activities she would do… you would hear kind of a wheezing kind of noise going on*”. Others reported seasonal change, “*like during the winter months*” or outdoor temperatures, “… *if it's um, real hot outside*” as potential triggers for their child’s wheeze. Wheezing incidents happened “*occasionally to a couple times a week*” for one caregiver’s child, to “*pretty much on a daily basis*” for another. Caregivers also described differing severities of wheeze, describing both milder cases and more severe, *“…it was just like, really, really heavy wheezing, like he was gasping for air”,* as mentioned by this caregiver of a 3 year-old boy.

Trouble breathing was another common symptom observed by 67% of caregivers (n = 10) and described in a variety of ways. “Difficulty breathing”, “shortness of breath” and “heavy breathing” were different descriptions used; for example, one caregiver explained “…*she might have really, um, heavy breathing… or at times she was having little shallow breathing*…* It seemed like*…* she couldn’t get enough air”.* For some caregivers, trouble breathing was observed on a “*daily basis*” while other caregivers noticed it “*maybe about once a month, um, or maybe once every other month”*. Certain triggers were noted, including seasonal changes, especially during “*winter*”, or when their child participates in sports or vigorous activities such as “*when he swims…when he’s really working very hard*”, or if their child has “*really bad colds or really bad viruses”*. In general, the symptom was easily observed, however, one caregiver of a 5 year-old boy realized her child’s behavior was a sign of his trouble breathing: “‘*What's wrong?’ He like, ‘Nothing, Nana, just laying here s- because I breathe better. Turn the fan on.’ Or, ‘Raise the window up.’ He asking for air. So then I have to give him a breathing treatment*”.

The most frequently mentioned impacts of symptoms on children were playing (n = 12), school (n = 9), and sleep (n = 8), reflecting the impact concepts (on play and sleep) in the PAQ. Asthma symptoms limited children’s ability to play and “run around” as described by this caregiver of a 4 year-old, “*At the playground… when I start seeing his face turn red and he, um, like startin’ to breathe heavily, we have to go, so, it's like he can’t play as long outside as the other kids*”. Caregivers described their child’s symptoms disrupting their school day, for example, by having to take medication, not being able to concentrate in class or participate in social activities, and missing school altogether. Disturbed sleep was mentioned by caregivers as a significant impact due to coughing or wheezing at night resulting in impaired learning, energy, and mood the next day. “*[His symptoms] causes him to not sleep well, so then the whole day that he is awake, and at daycare, and everything else, he's just super grumpy, super irritable, not really wanting to pay attention to the teacher and learn*”, explained a caregiver of a 4 year old boy.

Overall, the symptom and impact discussion with caregivers gave insight into the very young pediatric asthma experience. This portion of the interview confirmed the concepts found in the PAQ are in fact experienced by this age group; further, there were no missing concepts identified by caregivers. Following the brief discussion, the interview transitioned to the cognitive debriefing of the PAQ.

### PAQ cognitive debrief results

Items were well-understood by the majority of caregivers; response options, recall period, and reportability were deemed appropriate based on their child’s asthma experience. All caregivers reported that the questionnaire was comprehensive, easy to understand and complete. While caregivers generally thought the measure covered the key concepts of their child’s asthma experience, there was one exception. Four caregivers (26.7%) indicated that Item 2 (recent ER visits or hospitalization) was not currently relevant for their child’s severity of asthma. Of the 15 caregivers, 11 (73.3%) said that their child experienced no symptoms or mild symptoms of asthma over the past 7 days; therefore, a few of the caregivers had not visited an ER or hospital for their child’s mild asthma.

### Response options

The response scales used in the PAQ are primarily based on questions about *how often* each item symptom or impact concept was experienced by the child. Items 1, 3, 4 and 6 used either days, nights, or days within a month period, eg, 0 days, 1–2 days, 3–4 days (Items 3, 6), or, One day in the past month, two days in the past month, (Item 1). There were very few comments or suggestions among caregivers for changes to these response scales. Caregivers found no issues with the yes/no response options for Item 2 (recent ER visits or hospitalization). Lastly, Item 5 (limitations in physical activity), which used a response scale of Not at all limited, A little bit limited, Somewhat limited, Very limited and Extremely limited, was viewed favorably by all but one caregiver who felt that ‘somewhat’ and ‘very’ limited were fairly similar concepts.

### Recall period

The 2 recall periods that were debriefed, “during the past week” and “during the past month” had good overall comprehension. Caregivers clearly described thinking of the prior 7 days from the present (time of the interview) when considering their answers for those particular items and thought that the timeframe was appropriate for the question. When debriefing “the past month” for select items (oral steroid use and ER visits), similarly, all caregivers noted thinking of 30 days, rather than the calendar month, when considering their answers. The month timeframe was also deemed appropriate by caregivers for those particular items.

### Observer reportability

Caregivers were confident in their ability to remember information about their child’s asthma symptoms during “the past week” and exacerbations in “the past month”, in order to report on them accurately. Many caregivers described frequent and detailed communication with their child’s preschool teacher or daycare provider about their child’s condition and medication. Therefore, if needed, caregivers could easily answer items by checking in with the child’s teacher/daycare for this information.

Table 3 presents item-level findings for the PAQ along with participant suggestions for clarifying or modifying items, response options, and instruction.

**Table 3:** PAQ cognitive debrief findings and participants’ suggested edits

Item	Understanding (n/N^*^)	Relevance (n/N)	Response options appropriate (n/N)	Recall period suitable (n/N)	Observer reportability (n/N)	Participant suggested change and quote
General instruction	15/15	NA**	NA	NA	NA	NA
Item 1: oral steroids, past month	9/15	13/15	13/15	13/15	14/15	Clarify mode of administration for oral steroids (n = 4): “I would say, um, in the oral steroids, uh, I would not just give an example of the drug, but I would give an example of the method of administration. So, the- I would call out pill or nebulized”. – SP-003
Item 2: emergency room/hospitalizations, past month	15/15	11/15	15/15	15/15	15/15	NA
Weekly timeframe	14/14	NA	NA	NA	NA	NA
Item 3: breathing problems, past week	15/15	15/15	15/15	15/15	14/15	Replace “how often” with “how many days” to reflect concordance between item and response options (n- = 2); “… it's asking you to count days, not to count time. So, um … when you said how- uh, how often, um, you might just say how many days.” – SP-005
Instruction to continue	15/15	NA	NA	NA	NA	Bold important aspect of instruction, ‘please stop here’ to make more visible (n = 3): “I would just bold it like how you did the previous one.” – CH-004
Item 4: nighttime breathing problems, past week	15/15	15/15	14/15	15/15	14/15	NA
Item 5: limitations from breathing problems, past week	15/15	14/15	13/15	15/15	14/15	NA
Item 6: quick relief medications, past week	13/15	15/15	15/15	15/15	14/15	Replace “how often” with “how many days” to reflect concordance between item and response options (n- = 2); “[In the item] when you say how often, but then you’re asking about specific number of days [in the response options], um….. I mean, so just say like how many days in the last week [in the item]”.—SP-001

*n = Number of participant responding positively to questions on individual item debrief concepts; N = Total number of participants asked debrief question

**NA Not Applicable

### Item modifications

Three items were modified based on participant feedback. Responses for Item 1 suggested that the phrase “oral steroids” was interpreted differently by participants. Interviewers probed specifically on this issue and found that perceptions about the mode of administration of oral steroids were mixed. While the name of an oral steroid was given, “(eg, prednisone)”, the mode of administration was not provided. Four participants, when asked to describe their thoughts about what the phrase meant, included inhalers in their answer. Two participants stated liquid only and another two were not fully clear on the meaning. Therefore, only 9 of the 15 (60%) participants were considered to have full understanding of the item by answering as the item was intended and considered only liquid or pill forms of steroids (Table 4).

**Table 4:** Number of participants with differing interpretations of the phrase ‘oral steroids’

Liquid only	Liquid or pill	Liquid, pill or inhaler	Unclear
2	7	4	2

For this reason, a phrase clarifying the mode of administration, “(tablet, capsule, solution or suspension; does not include inhalers)” was added to the item to provide simple and clear guidance to caregivers about the types of oral steroids to include when answering the question.

Items 3 (breathing problems, past week) and 6 (quick relief medication use, past week), had similar item stem structures, asking caregivers “how often” their child had breathing problems, or used quick relief-type medications for asthma symptoms, respectively. The response scale presented 5 options in day format, e.g., 0 days, 1–2 days, etc. Two participants suggested using the phrase “how many days” in the item stem, rather than “how often” to align better with the day format of the response options. For example, one participant explained, “… *it's asking you to count days, not to count time. So, um *…* when you said how- uh, ‘how often’, um, you might just say ‘how many days’*” (SP-005). Both items were modified to reflect this change.

Minor comments or suggestions for improvements of the instructions (n = 4), response options (n = 4) and recall period (n = 2) were made, however, comments were not consistent across participants and suggested changes (eg, minor wording or phrasing preferences) were not necessary to support the content validity of the measure.

## Discussion

Each year, 1 in 6 children with asthma visits the ER. Poorly controlled asthma in children is the focus of a CDC 2019 nationwide initiative called CCARE (Controlling Childhood Asthma and Reducing Emergencies) [28]. The objective is to prevent ER and hospitalizations by 500,000 by 2024 by tracking children’s health and teaching evidence-based interventions. While the initiative addresses just one aspect of the growing childhood asthma problem, it also underscores the need for additional tools to monitor and prevent asthma worsening. The PAQ was developed to fill the need for a brief yet comprehensive caregiver-reported symptom control and asthma-specific HRQoL measure in mild-to-severe young asthma patients, by evaluating exacerbation control (2 items), severe exacerbations (1 item), symptom control (1 item), and asthma-specific HRQoL (2 items). Assessment of these domains within a single 6-item measure may allow the PAQ’s use to extend beyond clinical trial research into clinical practice and real-world studies. Significantly, the inclusion of shorter recall periods for the symptom/HRQoL items avoids a major limitation of some existing pediatric asthma measures that use recall periods of 4 weeks or more [16]. Indeed, after undergoing psychometric testing, the measure may be appropriate to use for tracking children’s health by caregivers or clinicians, for example, or to evaluate the efficacy of new asthma therapies in clinical trials or other types of research where measurement of asthma symptoms and asthma-related overall health of young children is of interest. In addition, the PAQ, which operates as an ObsRO rather than a proxy measure, in that the concepts assessed are strictly observable without any inference or assumptions by the caregiver (ie, PROMIS Parent Proxy Asthma Impact – Short Form 8a), is designed specifically for 2–5 year-olds where patient self-reported measures are suboptimal.

Qualitative data from caregivers about their observations of their child’s asthma experiences reflects the burden of their child’s symptoms, which can impact their child’s daily life. The key symptoms identified during the open-ended discussion portion of the interview, Coughing, Wheezing and Trouble breathing, map closely to the symptoms referenced throughout the PAQ, ‘coughing, difficulty breathing, or noisy breathing from the chest’. By analyzing the interviews in cohorts of n = 5 it was possible to show that there were no new symptom concepts emerging by the last set of interviews, indicating saturation of concepts [29] important to consider in this very young child asthma population. (Lethargy, nosebleed and watery eyes were mentioned by a single caregiver each in the last interview set, however as these symptoms are not specific to asthma and reported by 1 individual, the study team concluded that additional interviews would not have yielded additional new asthma concepts). This supports the inclusion of these 3 symptoms in the measure and confirms that the content of the measure accurately reflects the main symptoms experienced by this 2–5 year-old patient population. These findings are supported by recent research which identified the same key symptoms in children aged 6–11 with asthma [30] and have been found to be the most frequently experienced asthma symptoms by all age groups [3, 31].

The HRQoL items, which focus on impaired sleep and physical activity, are highly relevant impacts specific to asthma patients. When sleep is disturbed, significant impairments affect the child the following day including fatigue and diminished focus and cognition in school, and over a longer term, may be associated with anxiety and depression [32]. Similarly, not being able to run, play, or participate in sports or physical activities can affect a child’s physical, mental and social wellbeing [33]. Therefore, by assessing these two most proximal HRQoL asthma-specific impacts, the items are able to capture the true sense of the burden in this age group in a succinct way to allow ease of monitoring compared to use of other lengthier measures (eg, PedsQL).

In terms of debrief findings, overall comprehension of the items was very strong. With the exception of clarifying “oral steroids” in Item 1, no other significant comments or suggestions were raised about understanding and caregivers answered each item as it was intended, ie, there were no ‘incorrect’ or misunderstood answers given for any of the items. All instructions were easy to understand for caregivers as well, with only one formatting suggestion. Based on positive overall findings and the inclusion of mode of administration for “oral steroids” in Item 1, researchers concluded that additional interviews were unnecessary in this instance.

Feedback on the individual items within the PAQ suggested that all items were relevant to almost all participants, the one exception being Item 2 (recent ER visits or hospitalizations). The mild/very mild subset of the population did not think it was relevant based on recent experience with their child but all participants thought this was a relevant item when considering the likelihood that their child’s symptoms may escalate, at some point, to needing urgent medical intervention and so should be included. The inclusion of this item differentiates the PAQ from other control measures such as the TRACK. The TRACK also utilizes lengthy recall periods (eg, 4 weeks, 3 months) for items, which may be more suitable for a milder, less-symptomatic patient population in which symptom monitoring is not occurring regularly. FDA guidance clearly states that instruments with long recall periods which rely on memory “are likely to undermine content validity… Items with short recall periods or items that ask patients to describe their current or recent state are usually preferable” [21]. Thus, the PAQ provides coverage for mild to severe patients and may detect, in a shorter timeframe, worsening symptoms in need of attention.

One of the main advantages of the PAQ is its brevity, and therefore its ease of completion, which lends itself to ease of monitoring asthma control either in clinical practice or in research studies. Indeed, arguably, the combined brevity, targeted age group, and well-defined purpose of the PAQ may make it a viable option to deploy in an app-based, mobile Health (mHealth) solution. Digital health solutions that include a diary or questionnaire (such as the C-ACT) have been found to generally be associated with better asthma outcomes, including improved asthma control [34]. However, the C-ACT, a well-validated instrument for symptom control in children aged 4–11 years, has been critiqued for low sensitivity due to “considerable” overestimation of asthma control by both children and their caregivers [35]. It has also been found to be burdensome to complete each day by both parent and child [36]. The PAQ is distinct in that items were designed to capture observable behaviors and symptoms indicative of a child’s asthma status, which may help limit overestimation of asthma control within this younger age range. As the digital health technology space expands, the PAQ may offer a foundation for the construction of an mHealth solution in pediatric asthma symptom control measurement for the youngest patients while limiting bias due to symptom overestimation and burden of completion.

It is important to note that while equal numbers of children with mild, moderate, and severe clinician-rated asthma severity were enrolled, this sample had a majority of children (73%, as indicated by caregiver report of their child’s asthma severity over the last 7 days) with well-controlled asthma at the time of the interview. Based on the findings, this suggests that caregivers found the items to be relevant even when their child’s symptoms are less active. Finally, the discrepancy between the clinician and caregiver ratings of severity may have been due to the difference in the rating timeframe, ie, past 3 months versus past 7 days.

## Limitations

There are a few study limitations worth noting. First, the number of caregivers that participated in this study is a typical sample size for one-on-one, in-depth qualitative research; however, all participants were English speakers recruited from the US. Thus, multiple locations were used to maximize geographical diversity. Secondly, although there is a spread of age and gender characteristics amongst caregivers, those without a high school degree are not represented in our sample. This segment of the population is often more challenging to recruit and thus under-represented in studies with smaller sample sizes such as this. However, every effort was made to use simple language in the PAQ to ensure maximum comprehension by a range of education levels.

Among the child sample, while a range of childhood asthma severity levels were included, the population is predominantly a White and Black/African American non-Hispanic population; other ethnic groups, such as Asians or Native Americans, are not represented. Therefore, it would be prudent to confirm the cultural representativeness of these findings and any translations of the measure with appropriate populations [37]. Additionally, though there are a noticeable number of participants who report ‘maintenance’ OCS use, based on the overall severity of the sample and evidence from the interviews, it appears long-term use of OCS medication is minimal. Lastly, the sample consisted of children between the ages of 2 and 5 years; if researchers desired to use the PAQ in older age groups, more interviews would be necessary to confirm the validity in that age group.

Future studies will need to evaluate the psychometric properties of the PAQ, including internal consistency, reliability, construct validity, and responsiveness, which will be informative for determining the scoring algorithm for the measure. Additionally, what constitutes a meaningful change also needs to be defined to aid in interpretation of changes in scores.

## Conclusions

The PAQ is a newly developed 6-item ObsRO measure for pediatric asthma that evaluates both symptom control and asthma-specific HRQoL impact in one measure, in patients aged 2–5 years. Compared to other asthma measures for this age group, it was designed to be a brief yet comprehensive ObsRO that may better detect worsening of symptoms due to the shorter recall periods for most items. The findings from this research provide evidence that the PAQ reflects symptom concepts experienced by these youngest patients, and that each item is relevant and well-understood by their caregivers. Thus, the content has been found to be valid within this population. Once it has been psychometrically validated, the PAQ will be an appropriate instrument for inclusion in asthma studies to measure the effect of treatment for mild to severe pediatric asthma, or for monitoring of symptom control in other real-world or clinical settings.

## Data Availability

The datasets generated and/or analyzed during the current study are not publicly available but are available from the corresponding author on reasonable request.

## Abbreviations

FDA: Food and Drug Administration
HRQoL: Health-related quality of life
ObsRO: Observer-reported outcome
OCS: Oral corticosteroids
PAQ: Pediatric Asthma Questionnaire
PRO: Patient-reported outcome
TRACK: Test for Respiratory and Asthma Control in Kids
US: United States
